# Direct observation of titanium-centered octahedra in titanium–antimony–tellurium phase-change material

**DOI:** 10.1038/ncomms10040

**Published:** 2015-11-27

**Authors:** Feng Rao, Zhitang Song, Yan Cheng, Xiaosong Liu, Mengjiao Xia, Wei Li, Keyuan Ding, Xuefei Feng, Min Zhu, Songlin Feng

**Affiliations:** 1 State Key Laboratory of Functional Materials for Informatics, Shanghai Institute of Micro-system and Information Technology, Chinese Academy of Sciences, Shanghai 200050, China

## Abstract

Phase-change memory based on Ti_0.4_Sb_2_Te_3_ material has one order of magnitude faster Set speed and as low as one-fifth of the Reset energy compared with the conventional Ge_2_Sb_2_Te_5_ based device. However, the phase-transition mechanism of the Ti_0.4_Sb_2_Te_3_ material remains inconclusive due to the lack of direct experimental evidence. Here we report a direct atom-by-atom chemical identification of titanium-centered octahedra in crystalline Ti_0.4_Sb_2_Te_3_ material with a state-of-the-art atomic mapping technology. Further, by using soft X-ray absorption spectroscopy and density function theory simulations, we identify in amorphous Ti_0.4_Sb_2_Te_3_ the titanium atoms preferably maintain the octahedral configuration. Our work may pave the way to more thorough understanding and tailoring of the nature of the Ti–Sb–Te material, for promoting the development of dynamic random access memory-like phase-change memory as an emerging storage-class memory to reform current memory hierarchy.

## Abstract (web summary)

Ti-Sb-Te is a promising phase change memory material however its phase transition mechanism is poorly understood. Here, the authors use microscopic and spectroscopic techniques to show that titanium-centered octahedra play a major role in boosting the performance of Ti-Sb-Te based phase change memory.

## Graphical abstract

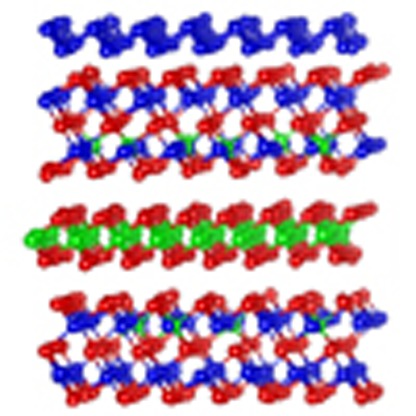

The performance gap between processor and memory has been widened in recent years since massively parallel computing with multicore processors being utilized^1^. An emerging storage-class memory (SCM) technology was introduced to mitigate the bottleneck, which concerns the entire computing system^1^,^2^. To reform the conventional memory hierarchy, the SCM technology needs to combine the attributes of memory and storage; that is to say, the performances of SCM are hoped to match commodity dynamic random access memory (DRAM), while the capacity and cost of SCM shall be able to compete with NAND Flash memory^1^. Phase-change memory (PCM) has been regarded as the most promising candidate to replace incumbent NAND Flash memory as well as DRAM for SCM application^1^,^3^,^4^. In terms of the speed, power, and endurance, the fact is PCM already has overall better bit performances than those of the NAND^5^. Obviously, multi-bits storage is indispensable to the evolution of a huge capacity NAND-like PCM. Hence, three-dimensional stacking of cells/arrays^6^,^7^ or multi-level resistance drift limitation in single cell^8^,^9^ will need a lot more work.

Nevertheless, the key challenges to realize DRAM-like PCM still lie in how to further decrease Reset operation energy and further increase Set operation speed, because DRAM requires rigorously 10^−11^–10^−12^ J per bit program energy and <10 ns access time^4^. The Reset operation of PCM, generally referring to the melt-quenching procedure, transforms the crystalline (c-) chalcogenide alloy, for example, Ge_2_Sb_2_Te_5_ (GST), into amorphous (a-) phase^10^,^11^. Conversely, the Set operation is to heat the a-phase to a temperature between crystallization temperature (*T*_c_) and melting point (*T*_m_) to obtain the c-phase^10^,^11^. Due to the slow crystallization speed (∼30 ns)^3^,^4^ and high *T*_m_ (∼616 °C)^12^ of GST-based materials, many efforts, such as diminish the grain size, promote the nucleation, reduce the active volume and enhance the thermal efficiency, have been made to try to lower the power and expedite the response^4^,^13^,^14^,^15^,^16^.

Our previous work proposed an alternative and simpler approach to develop DRAM-like PCM via merely replacing the switching material from GST to Ti_0.4_Sb_2_Te_3_ (TST) alloy^17^. We demonstrated at least one order of magnitude faster Set speed and as low as one-fifth of the Reset energy on TST-based PCM cell compared with those of GST cell with the same size^17^. Nevertheless, the origin of such performance improvements yet is controversial^18^ and not fully understood because of the lack of solid experimental evidences. In the present work, we apply Cs-corrected scanning transmission electron microscopy (STEM) and soft X-ray absorption spectroscopy (sXAS) combined with density functional theory (DFT) simulations to explore the phase transition mechanism of TST material in a more accurate way.

We report that the Ti-centered octahedra (TCOs) not only disperse inside of the hexagonal (HEX) Sb_2_Te_3_ (ST) lattice, but also form nano-lamellae separated outside, respectively acting as lattice distortion centers and nano-thermal insulators to induce low-energy amorphization of the restricted ST crystal grains. We also identify that in a-TST, the Ti atoms prefer to maintain the octahedral configuration. Such robust TCOs or nano-lamellae thus may serve as intrinsic nucleation centers or templates for surrounding Sb-centered atomic motifs to swiftly align into long-range order to achieve high-speed recrystallization. An understanding of the phase-transition mechanism of TST from the atomic and electronic structure points of view is essential for further material optimization to meet the requirements of future DRAM-like PCM application.

## Results

### Identification of triple-layered TiTe_2_ lamellae

**Figure 1:**
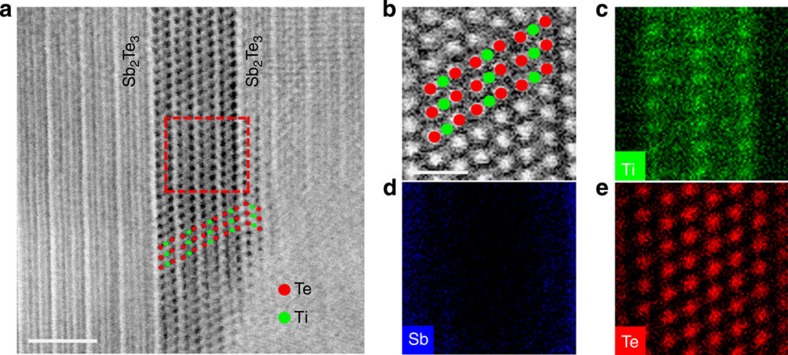
Structural and chemical identifications of TiTe_2_ lamellae in crystalline Ti_0.4_Sb_2_Te_3_. (**a**) Scanning transmission electron microscopy (STEM) bright-field image of crystalline Ti_0.4_Sb_2_Te_3_, projected along <100> direction. (**b**) STEM high-angle annular dark-field image zooming into the field marked in **a**. (**c**–**e**) Energy dispersive spectrometer mappings of Ti, Sb and Te, respectively. Green, blue, and red points correspond to Ti, Sb and Te atoms, respectively. Scale bar, (**a**) 2 nm and (**b**) 1 nm.

**Figure 2:**
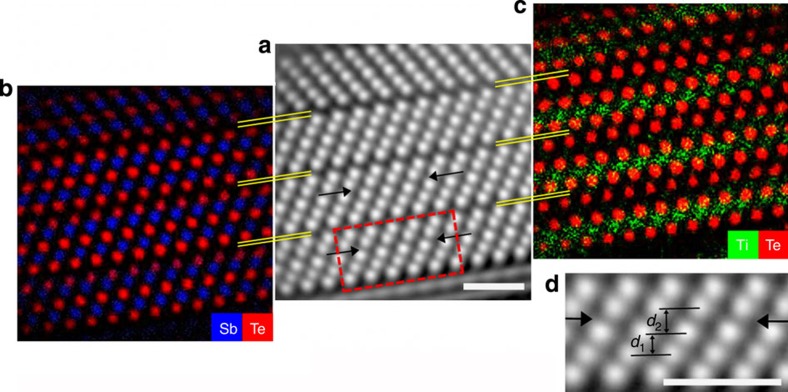
Structural and chemical identifications of quintuple layers in crystalline Ti_0.4_Sb_2_Te_3_. (**a**) Scanning transmission electron microscopy (STEM) high-angle annular dark-field (HAADF) image of quintuple layers, projected along <100> direction. (**b**) Energy dispersive spectrometer (EDS) mappings of Sb and Te. (**c**) EDS mappings of Ti and Te. (**d**) STEM-HAADF image zooming into the field marked in **a**. Compared with the interlayer distance (*d*_1_) between Sb(Ti) and Te_(2)_ layers, the wider gap (*d*_2_) between Te_(2)_ and Sb layers is pointed out with black arrows. The Te⋯Te Van der Waals interaction gaps are marked by double-line. Scale bar, (**a**,**d**) 1 nm.

From the STEM bright-field (BF) image of c-TST (Fig. 1a), several atomically resolved triple-layered lamellae can be clearly observed. They locate adjacent to the quintuple layers (QLs). Figure 1b shows the high-angle annular dark-field (HAADF) STEM image of the triple-layered lamellae zooming into the field marked by dashed line in Fig. 1a. The energy dispersive spectrometer (EDS) mappings of the Ti, Sb and Te elements for the corresponding zooming area in Fig. 1b are given by Fig. 1c–e, respectively. The triple-layered lamellae are determined to be composed with a stacking sequence of -v-Te-Ti-Te-v-Te-Ti-Te-v-Te-Ti-Te-v- (Fig. 1b and Supplementary Fig. 1), where v denotes the van der Waals interaction gap. Such stacking sequence is the typical HEX lattice structure of TiTe_2_ (TT) with lattice parameter *a*=3.77 Å (ref. 19). While the adjacent QLs are believed to be -v-Te-Sb-Te-Sb-Te-v- stacking structure of HEX- ST with a little larger lattice parameter *a*=4.25 Å (ref. 20), which can be confirmed in Fig. 2. Although TT and ST were commonly used to construct superlattice-like materials^21^, the ∼11% mismatch of *a* between TT and ST blocks still tends to result in the phase segregation. Differently magnified STEM-HAADF images and the corresponding EDS mappings of other observing regions in the same c-TST sample also confirm this segregation phenomenon that the ST crystal grains are shrunk or even cut into pieces by the TT lamellae (TTL) (Supplementary Figs 2 and 3). In contrast, the *a* values of GeTe (GT) and ST constructing the flagship GST alloy only deviate slightly (∼2.2% for HEX lattice and ∼1.0% for rocksalt lattice)^20^. Thus, unlike the incompatibility between TT and ST, it would be rare to find severe GT segregation out of ST in c-GST phase. Nevertheless, the smaller ST crystal grains defined by TT in c-TST may be helpful to boost the speed and power performances of PCM^14^.

### Identification of Ti-centered octahedra in Sb_2_Te_3_ quintuple layers

The HAADF image (Fig. 2a) shows the clear ST-like HEX lattice structure of the QLs in c-TST, which is atom-by-atom chemically identified as -v-Te_(1)_-Sb-Te_(2)_-Sb-Te_(1)_-v- stacking sequence (Fig. 2b), where subscripts (1) and (2), respectively, refer to 3-coordinated and 6-coordinated Te atoms. To be noted that there are non-ignorable Ti signals simultaneously detected in some Sb layers (Fig. 2c), which indicates that dopant Ti atoms can occupy Sb lattice sites in the HEX-ST lattice^17^. It is also worthy of pointing out that not all the QLs of ST in c-TST accommodate the Ti dopants (Supplementary Fig. 4).

## Discussion

**Figure 3:**
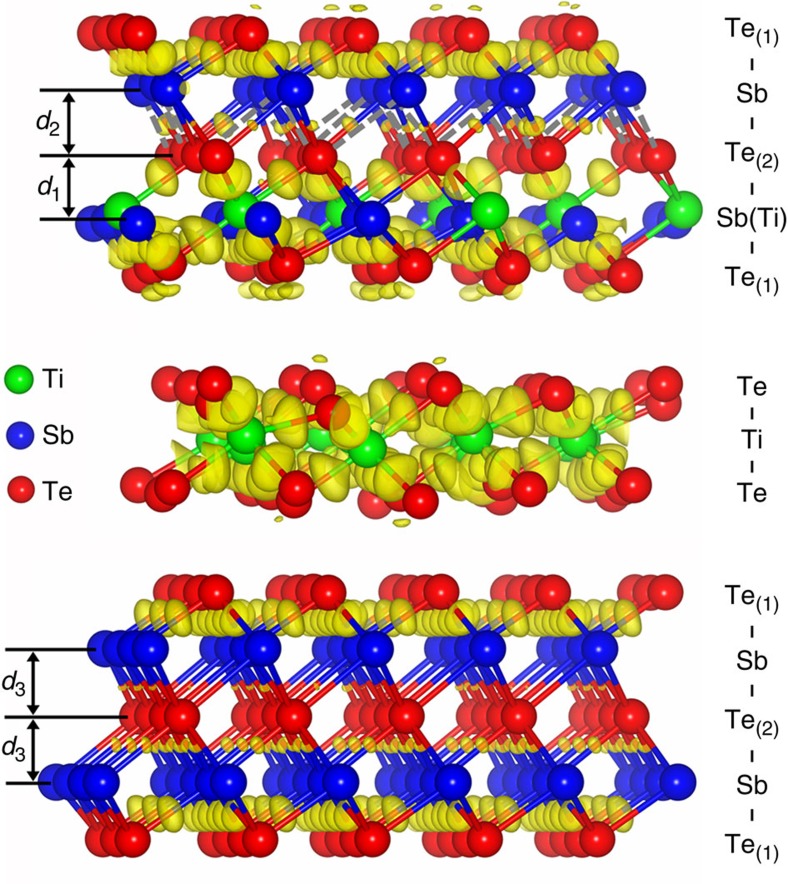
Lattice structure and bonding chemistry of crystalline Ti_0.4_Sb_2_Te_3_. Hexagonal lattice of Ti_0.4_Sb_2_Te_3_ with charge-density difference. The differences are calculated with respect to that of a superposition of isolated atoms. The isosurface (transparent yellow area) shows electron pileup mostly at the bonds. The isosurface value is fixed at +0.005 *e a*_*0*_^−3^ (*a*_*0*_=bohr). The Sb-centered octahedron has three strong bonds and three weak bonds, while the Ti-centered octahedron has six strong bonds and no weak bond. In quintuple layers (QLs) with the Ti dopants, the formation of strong Ti-Te_(2)_ bonds weakens some of the adjacent Te_(2)_-Sb bonds as marked by dashed lines, leading to a larger interlayer distance (*d*_2_) between Te_(2)_ and Sb layers compared with that (*d*_1_) between Sb(Ti) and Te_(2)_ layers. While in pure Sb_2_Te_3_ QLs, the interlayer distances (*d*_3_) among Sb-Te_(2)_-Sb layers are almost the same.

Given above, the representative lattice structure of HEX-TST should be depicted as the model presented in Fig. 3 from which, in addition to the pure ST QLs, one may also find the separated TTL as well as the TCOs scattered in ST QLs. The charge density difference^22^ maps indicate that in the Sb-centered octahedron, there are three strong and three weak bonds^23^; while among Ti and its six Te ligands in the TCO, there are six equally stronger covalent-like bonds formed by overlapping Ti-3*d* and Te*-*5*p* orbitals, which are quite similar with the electronic configuration of pure HEX-TT^24^,^25^. Due to the stronger Ti-Te_(2)_ bonds, the Te_(2)_-Sb bonds in the adjacent layer without the Ti are significantly elongated and weakened, as indicated in Fig. 3 by dashed lines. The rigid TCOs possibly bring tensile stress to the ST QLs, and those fragile Te_(2)_–Sb bonds have to be deformed to accommodate such lattice distortion. Thus, with more Ti atoms occupying in the same Sb layer, the interlayer distance (*d*_1_≈1.9 Å) between Sb(Ti) and Te_(2)_ layers is found to be a little smaller than that (*d*_2_≈2.1 Å) between the Te_(2)_ and Sb layers (Fig. 3). This subtle interspace expansion is also pointed out by black arrows in Fig. 2a,d. In comparison, there should be no such inter-distance variation for Sb–Te_(2)_-Sb layers (*d*_3_≈2.0 Å) in pure ST QLs (Fig. 3). During amorphization the fragile Te_(2)_-Sb bonds will be easily ruptured to destroy the long-range order, leading to a low-energy Reset operation. By contrast, the TTL with all rigid Ti-Te bonds may tend to keep its ordered configuration in amorphization, and in reverse effectively restrict the growth of ST grain. Since the quasi-two-dimensional c-TT is semimetallic^25^, and also has quite lower thermal conductivity (0.12 W mK^−1^)^26^ than those of c-GST (0.41 W mK^−1^)^27^ and c-ST (0.78 W mK^−1^)^28^, electric current shall be more conveniently conducted to the small ST grains to generate Joule heat that is insulated by adjacent TTL to accomplish a highly efficient Reset operation.

Comparing with our previous differential scanning calorimetry (DSC) result of TST material^17^, the remeasured DSC curve (Supplementary Fig. 5) expands the measuring temperature from ∼600 to ∼650 °C. Here we only focus on the melting procedures. The onsets of two endothermic peaks at ∼557 and ∼603 °C are taken as the first and the second *T*_m_s of TST material, respectively. Note that most of the heat is consumed by the second melting process which has a lower *T*_m_ than that of pure ST (∼618 °C)^12^,^29^. Chemically, we can rewrite the c-Ti_0.4_Sb_2_Te_3_ stoichiometry into (TiTe_2_)_x_Ti_0.4-*x*_Sb_2_Te_3−2*x*_ (0<*x*<0.4), where (TiTe_2_)_*x*_ part accounts for the TTL located outside of the ST QLs, then the rest Ti_0.4−*x*_Te_0.8−2*x*_ from Ti_0.4−*x*_Sb_2_Te_3−2*x*_ part should be considered as the TCOs inside of some ST QLs. Note that the Ti_0.4−*x*_Sb_2_Te_3−2*x*_ part (0<*x*<0.4) resembles the Sb-rich Sb-Te compound (2/3< Sb at.%/Te at.% <2/2.2) whose widely accepted microstructural fingerprint is the Sb_2_ bilayers stacking adjacent to some ST QLs^30^,^31^. Indeed, we also observed such structure of minority from individual crystal grains in c-TST (marked by black arrows in Supplementary Fig. 6). No doubt, this Sb-rich composition can naturally help to result in a faster crystallization speed than that of a-GST^30^. However, without accurately quantifying the value of *x*, we cannot determine the repeated stacking manner of Sb_2_ and ST blocks for Ti_0.4−*x*_Sb_2_Te_3−2*x*_ part, thus we prefer not to include the Sb_2_ bilayer in the representative lattice model of HEX-TST shown in Fig. 3. Furthermore, because pure TT material has quite larger *T*_m_ (>1,200 °C) than those of the Sb–Te compounds^29^,^32^, it is rational to deduce the TTL and TCOs could be unmelted during c-TST amorphization. Actually, our *in situ* X-ray diffraction results of pure TT film on Si substrate verify that even at 700 °C (>*T*_m_ of TST) (Supplementary Fig. 7), TT film still maintains good crystallinity with a typical HEX lattice structure^32^. Therefore, we believe the Ti_0.4−*x*_Sb_2_Te_3−2*x*_ part in TST play the major role in causing the two consecutive melting processes resembling the case of pure Sb-rich Sb–Te compounds^29^; especially the robust TCOs highly distort the HEX-ST lattice, promoting the significant decrease of the second *T*_m_.

On the other hand, in recrystallization, we speculate that the long-range ordered TTL and the structurally similar TCOs may serve as the heterogeneous nucleation templates or centers for neighbouring distorted or destructive Sb-centered octahedra to easily and swiftly align into long-range order^13^,^14^,^33^,^34^. This ordering mechanism is qualitatively different with the one of a-GST that involves a reconfiguration of considerable tetrahedrally bonded Ge atoms to octahedrally bonded ones^35^,^36^ or a cavity-supported reorientation of four-membered ring structures^37^,^38^. In this regard, we also note that in Si doped ST material, Si and ST exhibit nano-scale phase segregation morphology resembling that of TST; namely, dopant Si atoms keep in the a-phase outside of the ST-rich areas and do not participate in the phase transition^39^. Nevertheless, the a-Si phase cannot play the role of inducing swift recrystallization of the a-ST phase because of the complete lattice mismatch between a-Si and c-ST, leading to a sluggish Set operation^39^.

**Figure 4:**
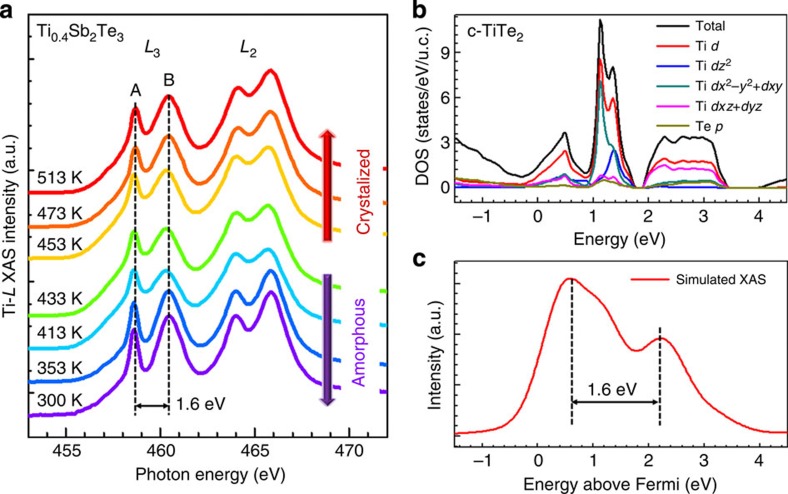
Octahedral configuration of Ti in both crystalline and amorphous Ti_0.4_Sb_2_Te_3_. (**a**) Ti *L-*edge soft X-ray absorption spectra of Ti_0.4_Sb_2_Te_3_ film measured at various temperatures crossing the a- and c-phases. The energy separation of double peaks (A and B) of *L*_3_ edge is sensitive to the splitting of Ti-3*d* orbitals resulting from the crystal field. The similarity of the temperature-dependent absorption spectra suggests the stable Ti-centered octahedral configurations keep the electronic structure of Ti unchanged. (**b**) The partial electron density of states of Ti in crystalline (c-) TiTe_2_ shows the splitting of Ti-3*d* orbitals. (**c**) The simulated Ti-*L*_3_ soft X-ray absorption spectrum of c-TiTe_2_ with electron-hole approximation. The good agreement of energy separation of double peaks in both calculated and experimental spectra suggests that the Ti-centered octahedral configurations in c-Ti_0.4_Sb_2_Te_3_ is very similar to the ones in c-TiTe_2_.

One may still inquiry whether the Ti atoms could keep their octahedral configurations even during high-temperature amorphization process. We thereby used sXAS method to verify the existence of TCOs in a-TST. The spot size of the incident X-ray beam for taking sXAS is about 1.5 mm^2^. In contrast to the STEM for microstructural probe, the sXAS technique can provide the overall information in macroscopic scale. For 3*d* transition-metal (TM) compounds, sXAS involves the excitation of 2*p* core electrons to empty 3*d* orbitals (2*p*^6^3*d*^n^ to 2*p*^5^3*d*^n+1^) through dipole selection rules^40^,^41^. Thus, sXAS directly probes the TM 3*d* unoccupied electronic structures that is sensitive to its oxidation states, spin states, and local environment such as chemical bonding, symmetry and metal–ligand distance. Figure 4a shows the Ti *L-*edge absorption spectra of TST film with 1 K min^−1^ *in situ* heating rate. Note that the as deposited TST film is amorphous, and will begin to be crystallized when the *in situ* heating temperature reaches the *T*_c_=451 K (Supplementary Fig. 8). The absorption spectra are divided into *L*_3_ and *L*_2_ regions, corresponding to 2*p*_3/2_ and 2*p*_1/2_ levels resulting from the 2*p* core-hole spin-orbital coupling, and further split to double-peak structure (labelled as A and B in Fig. 4a) by the crystal field generated by the Te atoms surrounding each Ti atom. To understand the effect of this crystal field on the electronic structure of TST, we perform the DFT calculation to analyse the electron density of states. Here we use HEX-TT (space group of *P*3*m*1 and point group of *D*_3*d*_)^32^ as a reference to analyse the sXAS spectrum taken at 513 K corresponding to the c-TST phase. In contrast to the well-studied analogue TiO_2_ with point group of *D*_4*h*_ (refs 42, 43), where the slightly distorted octahedron of O atoms leads the splitting of degeneracy Ti-3*d* orbitals into *t*_2*g*_ and *e*_*g*_ orbitals with an energy separation 10 *Dq*, the fivefold degenerate orbitals of HEX-TT are split into three parts: the upper two degenerate orbitals of 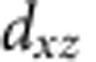 and 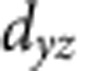, the middle orbital of 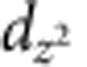, and the rest two degenerate orbitals of 
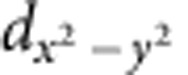
 and 
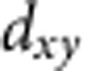
, as shown in Fig. 4b. The simulated Ti-*L*_3_ sXAS from electron-hole approximation is displayed in Fig. 4c, which shows the double peaks with energy separation about 1.6 eV. This energy scale quantitatively agrees with that of the experimental sXAS spectra for c-TST (Fig. 4a). Although the ratio of double-peak intensities is not well reproduced that may due to the lack of considering the electron-hole interaction^44^, this distinction is trivial for our study that focuses on the effect of crystal field. Because the temperature-dependent sXAS spectra, in particular the crystal-field separation, display almost the identical features, the Ti electronic configurations should be nearly unchanged in both a- and c-TST phases. This is the evidence, from the electronic structure point of view, proving that the Ti atoms maintain in the octahedral configuration in a-phase. Furthermore, our *ab initio* molecular dynamics simulation on melting TST alloy (data not shown here) also indicates that the ST QLs can be adequately disordered while the TTL basically preserve the ordered triple-layered structures. In this regard, we speculate that the separated TTL are able to survive after amorphization if the Reset operation of the TST PCM cell can be carefully manipulated. It has great potentials to design a suitable heating profile (<*T*_m_ of pure TT) to preserve the ordered TTL inside a disordered a-ST network. Higher-quality STEM and EDS evidences of ordered TTL formed in Reset state of the TST PCM cell still need more work.

In conclusion, STEM and sXAS studies of the TST material have revealed its essential microstructure feature that Ti atoms do exist in the octahedral configuration in both a- and c-phases. We have identified the dispersed TCOs in the HEX-ST QLs as well as the externally separated TTL. The former ones highly distort the ST lattice, while the latter ones effectively aggregate the heat in the small ST grains, both promoting the low-energy amorphization of c-TST. The octahedral configuration centered by Ti atom is proved to be prevailed in a-TST, which should originate from the strong covalent-like characteristic of Ti-Te bonds. The precise roles of the TTL and TCOs in triggering the swift recrystallization are not yet clear. We conjecture the long-range ordered TTL and the structurally similar TCOs may probably act as the medium for inducing the reorder of surrounding Sb-centered atomic motifs. The quantification of Ti contents in TTL and TCOs requires more study, and will be a great help to determining the lattice structure, understanding the phase transition mechanism and further optimizing the material composition for DRAM-like high-performance PCM application. In addition, the finding of intrinsic quasi-two-dimensional c-TT lamella in the polycrystalline TST alloy may open up the possibility of searching the topological insulating or superconducting properties from a lattice configuration well-controlled Ti–Sb–Te alloy^45^,^46^.

## Methods

### Film characterization

The composition of TST film was measured by X-ray fluorescence spectroscopy using a Rigaku RIX 2100 system. TST film was deposited on silicon substrate at room temperature, and annealed at 250 °C for 2 min to get the c-phase^17^. Cross-sectional view TEM sample of TST film was prepared by using Ar ion milling method. The atomic identification was carried out by using JEOL ARM 300F and FEI Titan 300 with probe-corrected STEM mode. *In situ* heating sXAS measurements of 150-nm thick TST film (deposited on silicon substrate at room temperature and covered by 2-nm thick Au film to avoid oxidization) were performed at beamline 20A1 of National Synchrotron Radiation Research Center at Taiwan. The beamline was equipped with a 6-m high-energy spherical grating monochromator to supply a photon beam with resolving power up to 8,000. The sXAS spectra were collected in total electron yield mode in an ultrahigh-vacuum chamber with a base pressure about 5 × 10^−10^ torr. All the spectra have been normalized to the photocurrent from the upstream clean gold mesh to eliminate the fluctuation of the beam flux. The photon energy was calibrated with the spectra of reference samples (SrTiO_3_) measured simultaneously.

### *Ab initio* theoretical simulation

The lattice model of c-TST was investigated by employing the DFT^47^. The Vienna *Ab initio* Simulations Package^48^ was used. The projector augmented wave pseudopotentials^49^ were used for electron-ion interactions. For the exchange-correlation energies between electrons, the Perdew-Burke-Ernzerhof (PBE) functional^50^ was used. A 216-atom supercell was built with 19 Ti, 65 Sb and 132 Te atoms. To reproduce the lattice structure of c-TST, a triple-layered TT was inserted between a Te⋯Te Van der Waals gap in HEX-ST and some Sb atoms were randomly replaced by dopant Ti atoms. The energy cutoff was set to 235 eV. The Γ point was chosen for relaxation and a 3 × 3 × 3 k-point mesh was used for electrical properties studies. In addition, sXAS simulation of c-TT used the all-electron full potential linear augmented plane wave plus local orbitals method^51^ as implemented in the Wien2k code^52^. The exchange correlation potential was calculated also using the PBE functional. The absorption spectroscopy was obtained through using a supercell calculation, where we removed a core electron on one atom in the supercell of 2 × 2 × 2, and added such electron into the conduction band.

## Additional information

**How to cite this article:** Rao, F. Direct observation of titanium-centered octahedra in titanium–antimony–tellurium phase-change material. *Nat. Commun.* 6:10040 doi: 10.1038/ncomms10040 (2015).

## Supplementary Material

Supplementary Information Supplementary Figures 1-8 and Supplementary References
