# Supplementary material for: Direct observation of titanium-centered octahedra in titanium–antimony–tellurium phase-change material
Source: Nat Commun. 2015 Nov 27;6:10040. doi: 10.1038/ncomms10040 (PMC4674681; doi:10.1038/ncomms10040)
Supplement: Supplementary Information — Supplementary Figures 1-8 and Supplementary References [file ncomms10040-s1.pdf]

Supplementary Figures

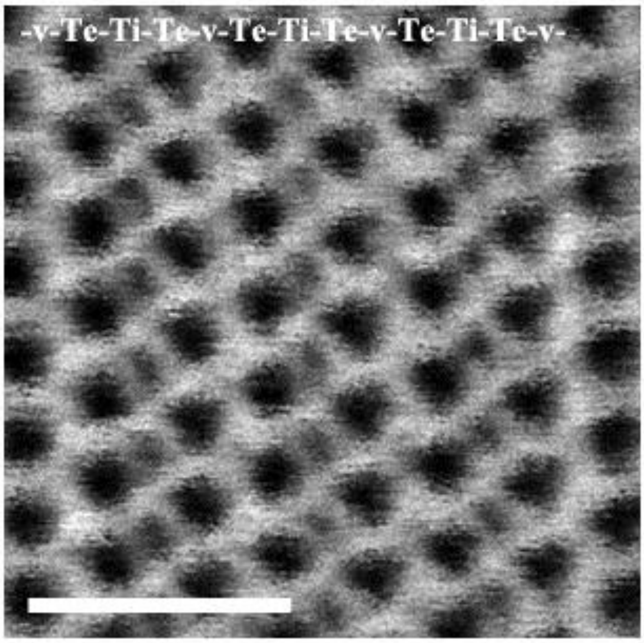

Supplementary Figure 1. Scanning transmission electron microscopy (STEM) bright field (BF) image of crystalline (c-) $Ti_{0.4}Sb_2Te_3$ (TST) zooming into the same field marked in Figure 1 (a) of the Main Text, projected along <100> direction. The scale bar corresponds to 1 nm. There are smaller black dots aligned and sandwiched between bilateral bigger black (darker) dots. Combined with the STEM high angle annular dark field (HAADF) image and energy dispersive spectrometer (EDS) results shown in Figures 1b-1e of the Main Text, the existence of -v-Te-Ti-Te-v- triple-layered structure is determined, where v denotes the Van der Waals gap. Because Ti has relatively smaller atomic radius and lighter atomic mass than those of Sb and Te, the smaller dark dots should be considered as Ti atoms in BF image, while the bigger and darker dots belong to the Te atoms.

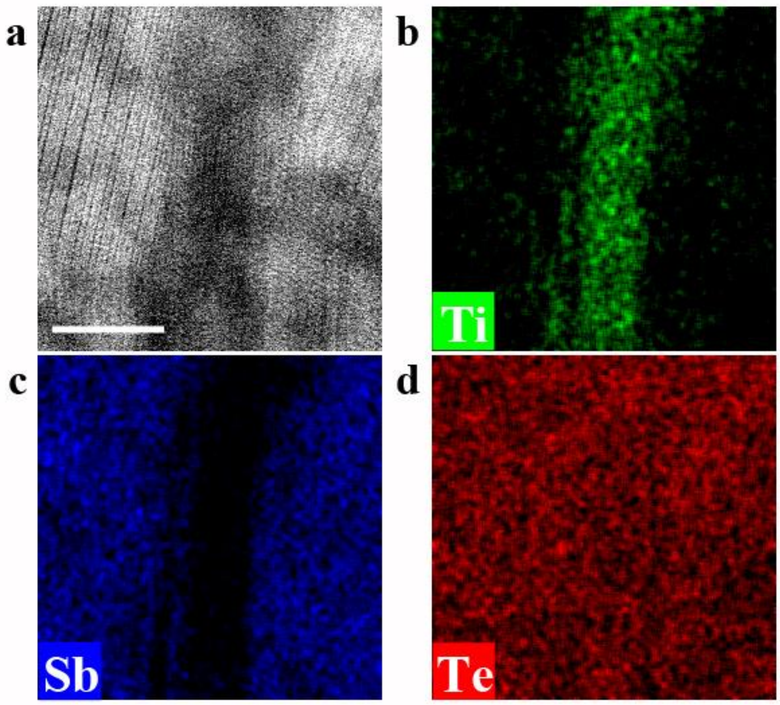

Supplementary Figure 2. Chemical identifications of c-TST. (a) STEM-HAADF image of c-TST. The scale bar corresponds to 10 nm. (b-d) EDS mappings of Ti, Sb, and Te, respectively. It is obvious that there is a rich area of Ti-Te compound located on the boundary between two Sb-Te crystal grains. And some scattered Ti signals can be observed inside the Sb-Te grains denoting Ti dopants can also enter into Sb_2Te_3 (ST) lattice.

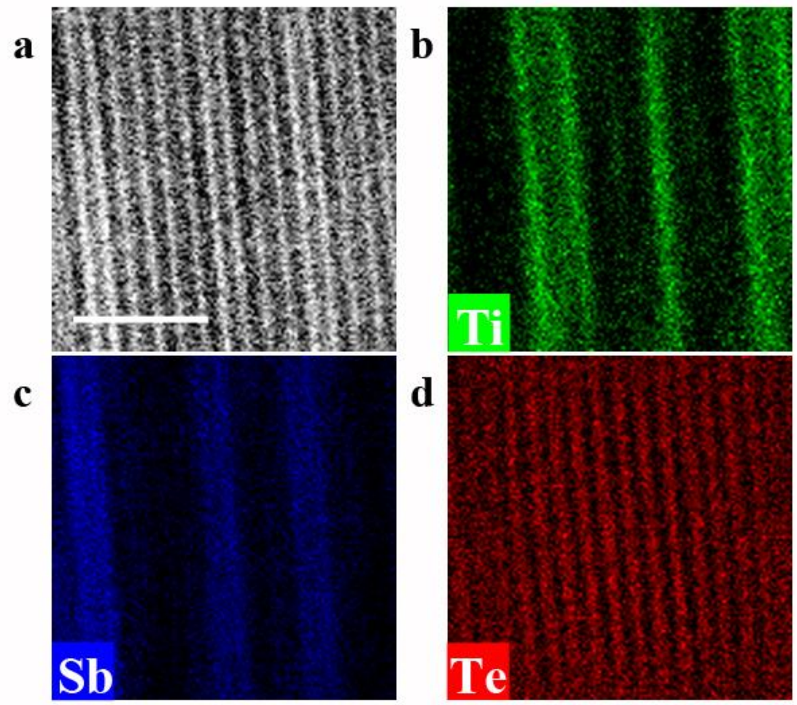

Supplementary Figure 3. Chemical identifications of c-TST. (a) STEM-HAADF image of c-TST. The scale bar corresponds to 2 nm. (b-d) EDS mappings of Ti, Sb, and Te, respectively. Comparing to the results shown in Supplementary Figure 1, the bigger magnification results here further confirm the nano-scale phase segregation between Ti-Te and Sb-Te compounds. In addition to the scattered distribution of Ti atoms in Sb-rich areas, most of the Ti atoms incline to accumulate into the lamella-shape forming the Ti-Te rich areas.

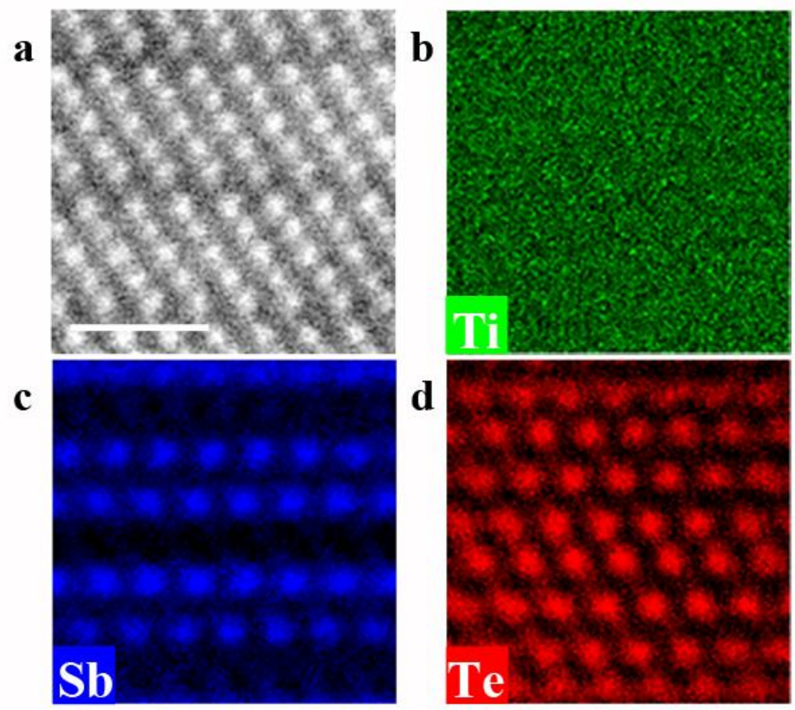

Supplementary Figure 4. Structural and chemical identifications of quintuple layers (QLs) in c-TST, projected along <100> direction. (a) STEM-HAADF image of the QLs. The scale bar corresponds to 1 nm. (b-d) EDS mappings of Ti, Sb, and Te, respectively. In contrast to the results shown in Figure 2 of Main Text, there is no Ti signal in the whole observation region or obvious inter-distance variation among Sb-$Te_{(2)}$-Sb layers, both denoting a pure hexagonal (HEX) lattice of ST.

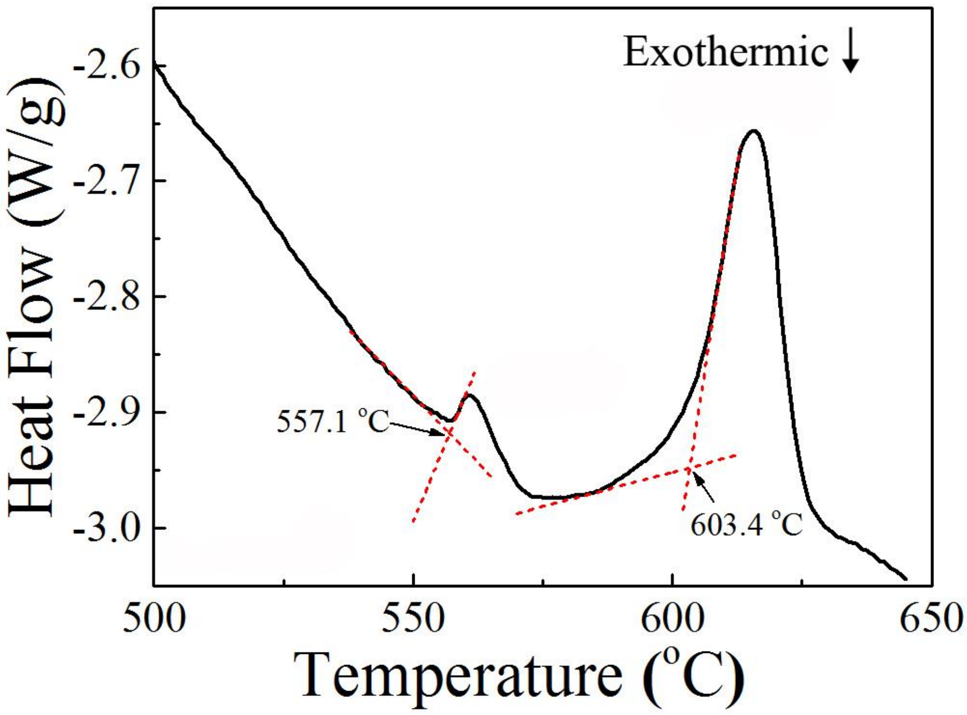

Supplementary Figure 5. Differential scanning calorimetry (DSC) curve of TST material verifying the two consecutive melting procedures. The heating rate is 10 °C min^{-1}. In contrast to the single endothermic peak at ~543 °C from our previous result[1], current DSC curve presents two consecutive melting procedures at ~557 °C and ~603 °C.

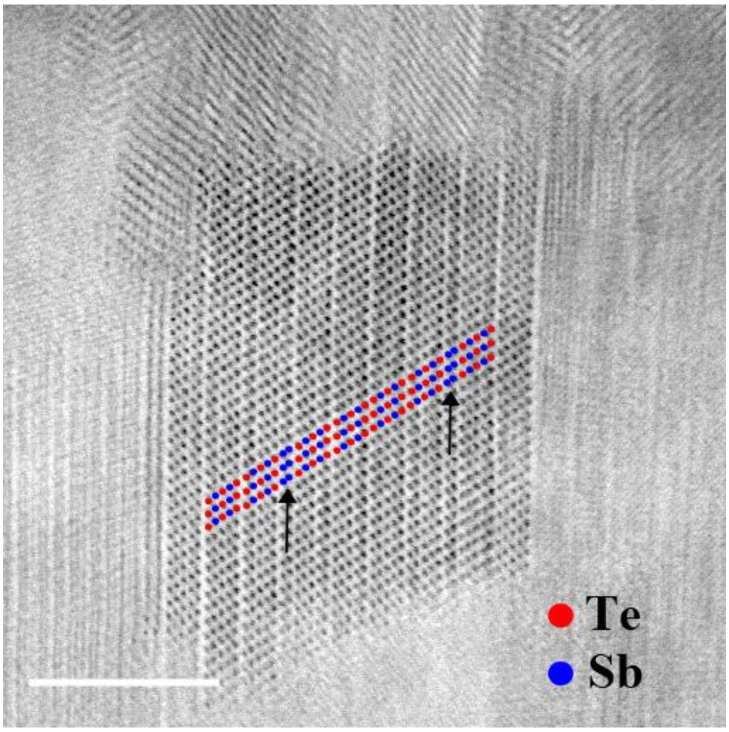

Supplementary Figure 6. STEM bright field image of a ST grain in c-TST, projected along <100> direction. The scale bar corresponds to 5 nm. Blue and red points correspond to Sb and Te atoms. The quintuple-layered structure of HEX-ST lattice can be clearly observed. Further, one can also find the seven-layered structure with the Sb_2 bilayer (marked with black arrows) adjacent to the ST QLs[2].

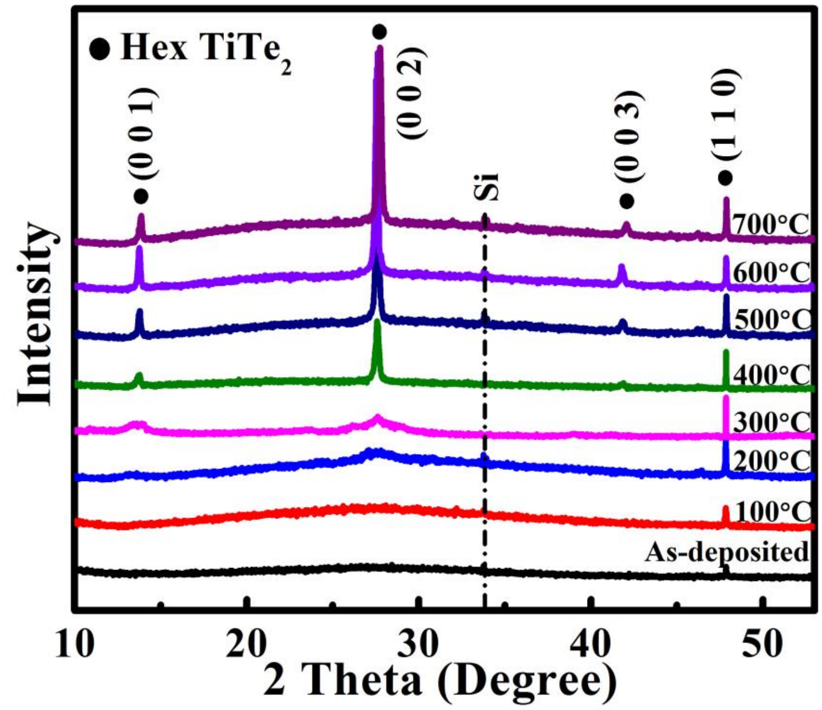

Supplementary Figure 7. *In situ* X-ray diffraction curves of 300 nm-thick pure $TiTe_2$ (TT) film on Si substrate. The as deposited TT film has already been partially crystallized. Even at 700 °C, the TT film still maintains the HEX lattice structure[3], which indicates the TT lamellae in c-TST cannot be melted in amorphization of TST material. Namely, the melting-quenched amorphous phase of TST should be mainly contributed by the amorphous Sb-Te compounds.

a

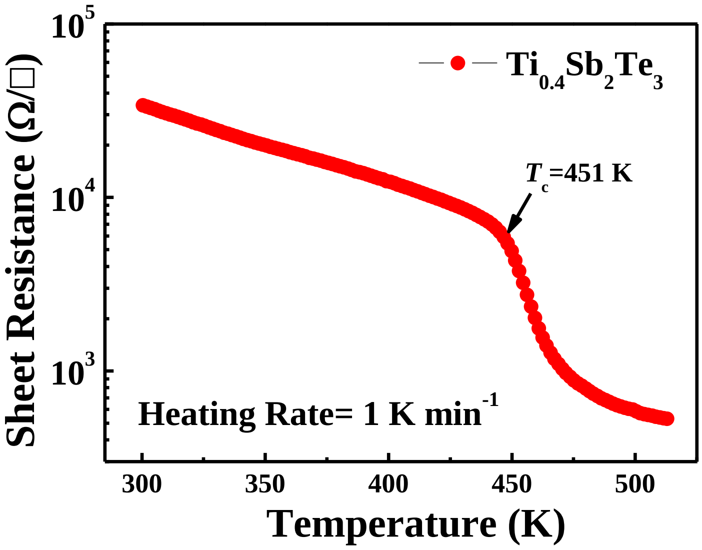

b

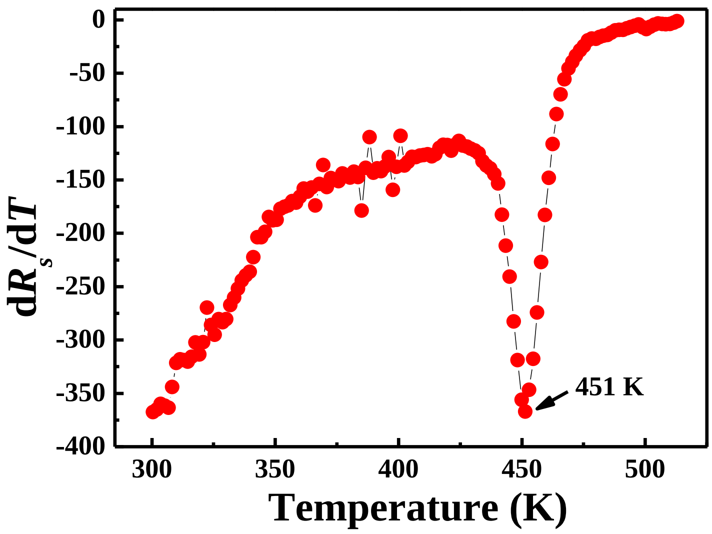

Supplementary Figure 8. (a) The variation of sheet resistance R_s as a function of temperature T of the as deposited 150 nm-thick TST film measured with *in situ* heating rate $\mathrm{d}T/\mathrm{d}t$ = 1 K min^{-1}, and (b) its first differential $\mathrm{d}R_s/\mathrm{d}T$ which reaches a minimum at 451 K. As the annealing temperature increases, a continuous decrease in R_s can be observed in (a). A sudden drop of R_s occurs ($\mathrm{d}R_s/\mathrm{d}T$ at the minimum)[4] when temperature reaches the crystallization temperature T_c = 451 K.

Supplementary References

1. Zhu, M. *et al.* One order of magnitude faster phase change at reduced power in Ti-Sb-Te. *Nat. Commun.* **5**, 4086 (2014).
2. Kifune, K. *et al*. Crystal structures of X-phase in the Sb–Te binary alloy system. *Cryst. Res. Technol.* **48**, 1011–1021 (2013).
3. Cordes, H. & Schmid-Fetzer, R. Phase equilibria in the Ti-Te system. *J. Alloys Compd.* **216**, 197–206 (1994).
4. Ryu, S. W. *et al*. Phase transformation behaviors of SiO_2 doped $Ge_2Sb_2Te_5$ films for application in phase change random access memory. *Appl. Phys. Lett.* **92**, 142110 (2008).
